# School’s In for Summer

**DOI:** 10.1289/ehp.113-a94

**Published:** 2005-02

**Authors:** John Manuel

While many of her classmates were waiting tables at restaurants during the summer of 2004, Alicia Smith was in an NIEHS lab investigating how exposure to sodium arsenite regulates the procarcinogenic and proinflammatory enzyme cyclooxygenase 2. Smith, then a rising senior at Orange High School in Hillsborough, North Carolina, is one of hundreds of high school, undergraduate, and graduate students nationwide who have participated in a paid summer student research fellowship supported by the NIEHS or the Community Outreach and Education Program of one of a number of NIEHS centers around the country. According to the sponsors and participants, these fellowships play an important role in exposing students to real-world science and encouraging them to pursue careers in environmental and biomedical research or health care.

## Summers of Discovery

The NIEHS Summers of Discovery program was launched in 1989 as a way to give talented high school, undergraduate, and graduate students, as well as high school and college faculty, a more in-depth exposure to the world of scientific research. Each year the program accepts 40–60 people from a pool of as many as 1,000 applicants. Participants are selected by scientific mentors from the NIEHS Division of Intramural Research according to the applicants’ area of interest and experience. Together they design and construct a research project to be carried out over a period of 8–12 weeks. Participants are paid a salary based on their level of education and experience.

At Summers of Discovery, students are exposed to the latest biochemical, molecular, and analytical techniques in their chosen field. Activities are usually done in conjunction with ongoing research at the NIEHS. The research is supplemented by a series of weekly seminars at which institute scientists present overviews of their work. At the end of the summer, students participate in a poster session where they display the results of their research and respond to questions as though they were at a national scientific society meeting.

“The work these students do most definitely furthers research at the NIEHS,” says Charle League, the institute’s coordinator of Summers of Discovery. “It’s a way to excite up-and-coming students about science and to grow the future research pool. Some end up staying on [at the NIEHS] to work part-time or return at a later date as full-time employees.”

Mike Humble, now a health science analyst with the NIEHS Division of Extramural Research and Training, participated in the program for two summers as a high school chemistry teacher. “Summers of Discovery was a tremendous experience for me,” Humble says. “It gave me a new perspective on how science applies to real-world research. When I went back to the classroom, I made sure my students understood why they were doing what they were doing. I taught them how to troubleshoot when things went wrong in their research.”

Faculty researchers who participate at Summers of Discovery say the experience benefits them as well. Joan Roberts, a professor of chemistry at Fordham University in New York City, points to two valuable aspects of the program. “First, it has been essential to my research career,” Roberts says. “I simply would not have been able to conduct the level of research in macular degeneration and cataracts without the equipment and support of the Laboratory of Pharmacology and Chemistry at NIEHS. Second, my undergraduate students in both the sciences and liberal arts have directly benefited from my collaborative research projects and my knowledge of cutting-edge research gleaned from seminars conducted during the Summers of Discovery. My students, although initially handicapped [by a lack of science background], have been accepted and successfully completed graduate science and medical programs in the top universities in the U.S. as a direct result of their undergraduate research program aided by the NIEHS Summers of Discovery program.”

## Marine Studies at Mount Desert Island

Maine’s Mount Desert Island Biological Laboratory (MDIBL)—which houses the Center for Membrane Toxicity Studies, one of the NIEHS Marine and Freshwater Biomedical Sciences Centers—offers summer research fellowships to approximately 12 high school students and 25 undergraduates each year. The high school students come from Maine, while undergraduates are recruited from all over the nation. Most are from small colleges or mid-sized universities that don’t have strong science programs. Half of the out-of-state students are racial minorities, a group that historically has been underrepresented in the scientific profession.

Started more than a century ago as a summer research program for the Tufts University School of Biology, MDIBL now operates as an independent, year-round research laboratory. In summer, the lab’s 7 full-time investigators and more than 50 seasonal investigators from around the country mentor the summer interns. Out-of-state students, who are funded by the National Science Foundation, focus on studying marine molecular physiology. Maine students, who are funded through the NIH and the Center for Membrane Toxicity Studies, study functional genomics in marine species. Examples of research projects have included factors of neurogenesis in the American lobster and transcriptional factor expression in the kidney of the adult skate.

As with Summers of Discovery, students at MDIBL attend seminars given by scientists who speak about their specific areas of research. High school students receive four days’ training at the beginning of the program to place their research in a broader scientific context. At the end of the session, students present their findings using PowerPoint slides.

“For high school students, the greatest benefit is that it opens a window to biomedical research that they may not get in school,” says Michael McKernan, director of education at MDIBL. Most high school science classes aren’t equipped to do molecular biology or study gene expression, he explains. Further, the program helps students plan their future by exposing them to teachers from different colleges where they might apply.

“For college undergrads, the program helps build their scientific repertoire in preparation for grad school,” McKernan adds. “They can get listed as coauthors on peer-reviewed papers. They get good recommendations for advanced degrees or for a job.”

## Farm Health in the Heartland

In America’s heartland, the University of Iowa’s Environmental Health Sciences Research Center funds the Environmental Health Sciences Institute for Rural Youth (EHSI). Each summer 15 rising high school sophomores from small communities in Iowa participate in this one-week full-scholarship residential research program. (EHSI will be expanded for summer 2006 to 25 students from Nebraska, Illinois, Missouri, and Kansas as well as Iowa if the center is able to identify additional funding to augment the existing program.) The research focus for EHSI students is on environmental health problems affecting rural communities, such as waste from industrial hog farms, long-term exposure to pesticides and fertilizers, and the effects on lung function of inhaled grain dust. Students benefit from a variety of learning activities, including didactic exercises, small-group discussion, and laboratory and field activities.

Nancy Newkirk, coordinator of the Environmental Health Sciences Research Center, says participating students gain critical exposure to science that leads many to pursue science education in college. “They have declared majors in such fields as biology, horticulture, global health medicine, agricultural systems technology, agronomy, wildlife, and forensic science,” she says.

Newkirk notes that 2005 will be the first year any former EHSI students will have completed their undergraduate education, “and that will give us a picture of their graduate school and career choices.” Newkirk hopes some of these students will choose careers in environmental science and return to rural farming communities to contribute their knowledge about environmental health.

## Cancer Research in Central Texas

The NIEHS Center for Research on Environmental Disease at the University of Texas M.D. Anderson Cancer Center, Science Park Research Division, offers its Summer Undergraduate Research Program (SURP) with yet another focus. M.D. Anderson is one of the nation’s leading comprehensive cancer research centers, and the program at Science Park focuses on providing undergraduate interns with a chance to participate in cutting-edge biomedical research related to environmental causes of cancer. Local high school students can also participate in a related program paralleling the SURP.

Typically, the summer program includes 15–18 undergraduates from all over the United States and Puerto Rico, and 4–6 high school students from the communities of nearby Bastrop and Travis counties. Summer intern research is supported by the center and by individual faculty research grants. In addition, an NIEHS training grant supports 10 minority undergraduate interns per year. The student programs include 10 weeks of hands-on bench research along with weekly scientific seminars, field trips, social events, and a final symposium at which interns deliver oral presentations on their work.

“The experience gained through training as a summer intern at the M.D. Anderson Cancer Center has not only provided me with the technical skills required for an occupation in the laboratory, but also the intellectual stimulation necessary to pursue a higher-level education as a research scientist,” says Nora Sanchez, a recent SURP intern. “Being that cancer research is the area I plan to pursue during my graduate studies, the center’s specialization in cancer has served as the foundation of my career.” Sanchez is currently applying to graduate school with plans to become a biomedical research scientist.

The center also offers a paid summer fellowship for three to five teachers of kindergarten through twelfth grade. Teachers of any subject can work at the center for four to six weeks, rotating through center facility cores, including molecular biology, cell analysis, and histology. In addition to learning about research techniques and goals, teachers also help the staff of the center’s Community Outreach and Education Program translate research findings into age-appropriate educational materials, including an environmental health web-site for students in grades 4 through 8 (http://www.veggie-mon.org/) and the Student Cancer Research and Evaluation Module, a molecular genetics site for high school students (http://www.scream.mdanderson.org/).

“My summer spent at Science Park was a great experience for me and translated into direct hands-on classroom activities for my middle school students,” says Elissa Quintero Adams, a seventh-grade science teacher at Cedar Creek Middle School in Bastrop. “It was easy and fun to include activities and information I gathered as an intern into my classroom.”

## Tomorrow’s Scientists Today

New skills, new perspectives, networking with mentors and peers—it’s an invaluable mix for students of any age. Summer research fellowships supported by the NIEHS offer students and teachers a tremendous learning opportunity as well as a chance to make money. But the payoff doesn’t end there. These students’ summer experience directly benefits ongoing scientific research at the host institutions and benefits the public at large by nurturing the next generation of scientists and medical practitioners.

## Figures and Tables

**Figure:**
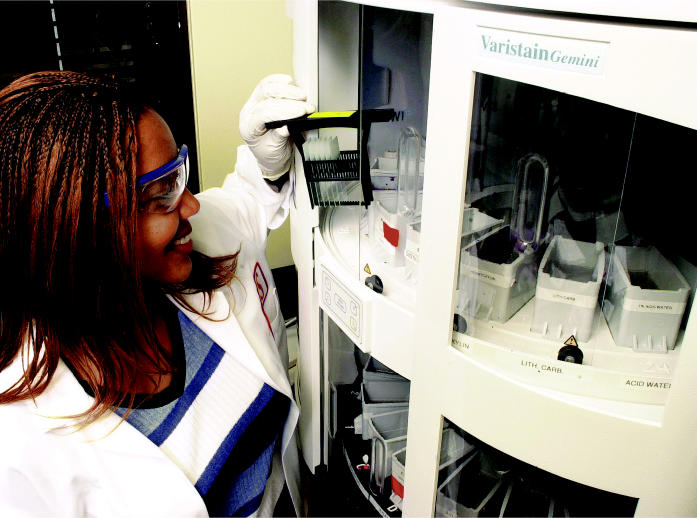
**No ordinary summer job.** The Summers of Discovery program at the NIEHS offered Marcia Sutton the chance to build her investigative skills in the Laboratory of Experimental Pathobiology.

**Figure:**
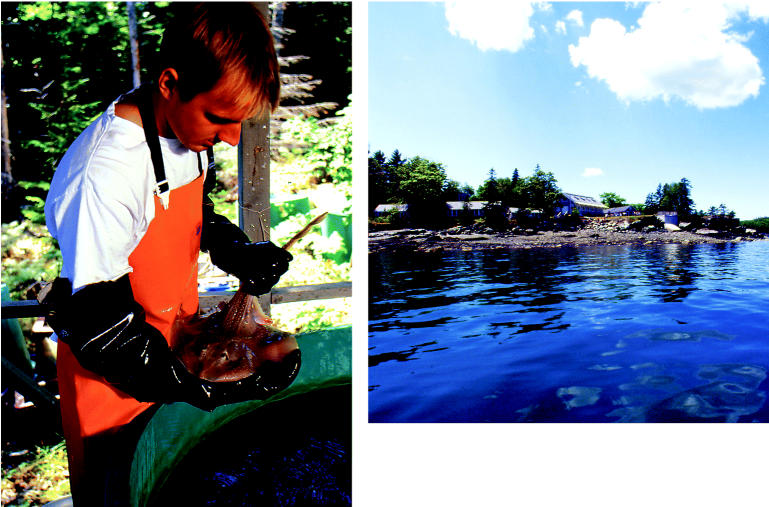
**Summer on the beach.** Former summer research fellow Davin O'Connell (left) examines a little skate—a marine model for studying xenobiotic transport—at the Mount Desert Island Biological Laboratory (above).

**Figure:**
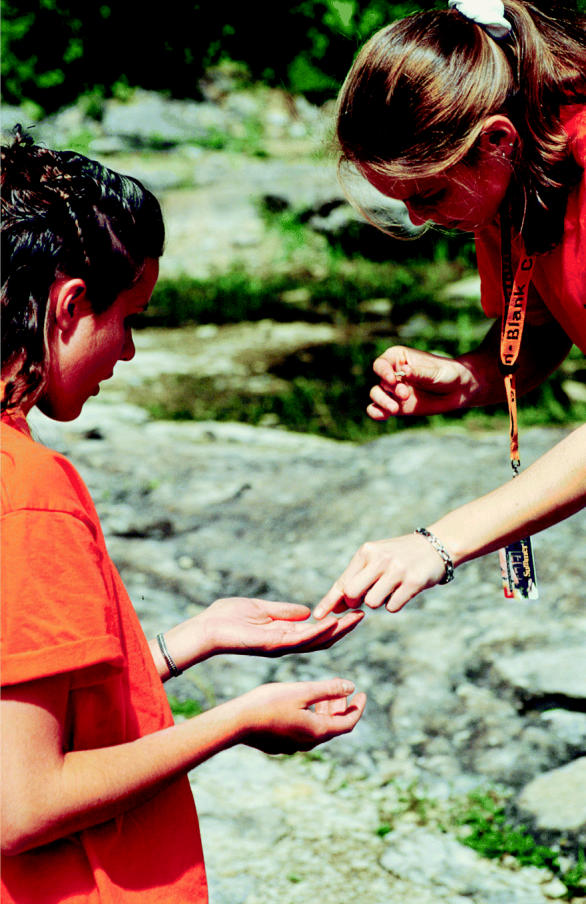
**Summer rocks!** Students in the Environmental Health Sciences Institute for Rural Youth spend some after-hours time examining fossils exposed by flooding at the Devonian Fossil Gorge in Coralville, Iowa.

**Figure:**
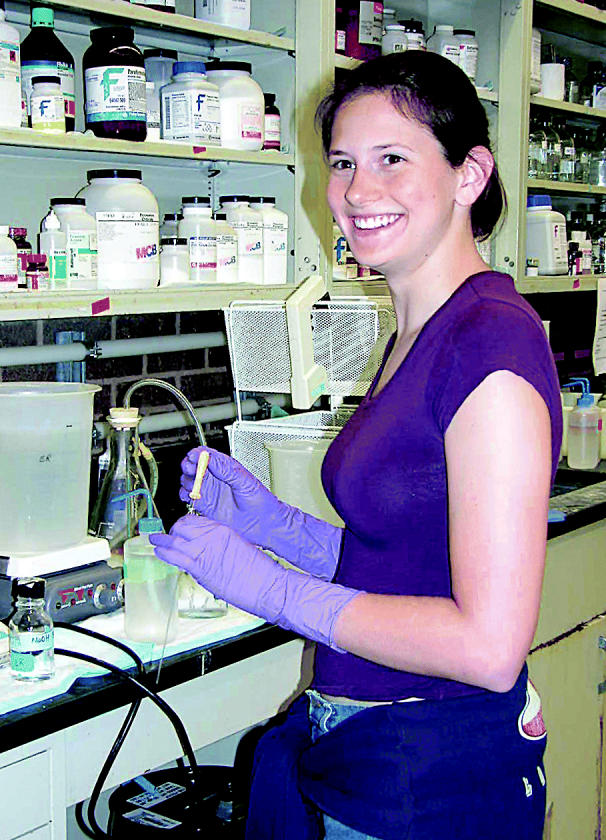
**Summer break from boring.** Jessica Ziaja gets hands-on lab experience through the Summer Undergraduate Research Program at M.D. Anderson Cancer Center, Science Park.

